# Supplementary material for: Integrating Inflammatory and Epigenetic Signatures in IBD-Associated Colorectal Carcinogenesis: Models, Mechanisms, and Clinical Implications
Source: Int J Mol Sci. 2025 Sep 28;26(19):9498. doi: 10.3390/ijms26199498 (PMC12524939; doi:10.3390/ijms26199498)
Supplement: Supplementary file 1 [file ijms-26-09498-s001.zip › Proofread_Supplementary Material_final.pdf]

Integrating Inflammatory and Epigenetic Signatures in IBD-Associated Colorectal Carcinogenesis: Models, Mechanisms, and Clinical Implications

Kostas A. Triantaphyllopoulos[1]*, Nikolia D. Ragia[1], Maria-Chara E. Panagiotopoulou[1], Thomae G. Sourlingas[2]

[1]Department of Biotechnology, School of Applied Biology and Biotechnology, Agricultural University of Athens, 11855 Athens, Greece
[2]Laboratory of Nuclear Proteins and Chromatin Function, Institute of Biosciences and Applications, National Center for Scientific Research "DEMOKRITOS", 15310 Agia Paraskevi, Greece.

* Corresponding author: Department of Biotechnology, School of Applied Biology and Biotechnology, Agricultural University of Athens, 75 Iera Odos St. 11855 Athens, Greece, e-mail: ktrianta@aua.gr

This **supplementary material** file provides additional data and visualizations supporting the main manuscript, offering deeper insight into the mechanisms linking inflammation to colorectal carcinogenesis. Included are extended gene expression analyses, epigenetic modifications, and non-coding RNA networks that complement the primary figures and tables. Specifically, the file contains:

TABLE OF CONTENTS **page**

Tables
Table S1.. 2
Table S2.. 8
Table S3..9
Table S4..13
Table S5..16
Table S6..18

Figures
Figure S1..20
Figure S2 21
Figure S3 22

ncFANdb 3.0 network input data..23

TABLES

Table S1. LncRNA - miRNA interactions inferred by ncFANs-NET network analysis in ncFAN v2.0 database(#) from human input data in Crohn's Disease (CD).

Gene_ID	Transcript_ID	Gene_Name	miRNA_ID	MRE_Count (§)	MRE_Location (§)	Max_miRANDA_score (*)
ENSG00000270084	ENST00000602767	*GAS5-AS1*	*hsa-miR-548ar-3p*	1	137-157-151.00|	151
ENSG00000270084	ENST00000602767	*GAS5-AS1*	*hsa-miR-582-3p*	1	307-331-160.00|	160
ENSG00000270084	ENST00000602767	*GAS5-AS1*	*hsa-miR-6745*	1	4-26-158.00|	158
ENSG00000270084	ENST00000602767	*GAS5-AS1*	*hsa-miR-5582-3p*	1	135-157-150.00|	150
ENSG00000270084	ENST00000602767	*GAS5-AS1*	*hsa-miR-625-3p*	1	438-459-164.00|	164
ENSG00000270084	ENST00000602767	*GAS5-AS1*	*hsa-miR-5006-5p*	1	93-113-150.00|	150
ENSG00000270084	ENST00000602767	*GAS5-AS1*	*hsa-miR-4646-5p*	1	462-483-160.00|	160
ENSG00000270084	ENST00000602767	*GAS5-AS1*	*hsa-miR-16-2-3p*	1	582-602-150.00|	150
ENSG00000270084	ENST00000602767	*GAS5-AS1*	*hsa-miR-19b-1-5p*	1	184-206-151.00|	151
ENSG00000270084	ENST00000602767	*GAS5-AS1*	*hsa-miR-301b-3p*	1	585-606-153.00|	153
ENSG00000270084	ENST00000602767	*GAS5-AS1*	*hsa-miR-1915-3p*	1	291-310-157.00|	157
ENSG00000270084	ENST00000602767	*GAS5-AS1*	*hsa-miR-363-5p*	1	6-26-162.00|	162
ENSG00000270084	ENST00000602767	*GAS5-AS1*	*hsa-miR-452-3p*	1	709-733-154.00|	154
ENSG00000270084	ENST00000602767	*GAS5-AS1*	*hsa-miR-195-3p*	1	580-602-150.00|	150
ENSG00000270084	ENST00000602767	*GAS5-AS1*	*hsa-miR-574-5p*	1	747-770-159.00|	159
ENSG00000270084	ENST00000602767	*GAS5-AS1*	*hsa-miR-6884-5p*	2	14-37-140.00|467-487-166.00|	166
ENSG00000270084	ENST00000602767	*GAS5-AS1*	*hsa-miR-8082*	1	496-521-154.00|	154
ENSG00000270084	ENST00000602767	*GAS5-AS1*	*hsa-miR-6864-3p*	2	103-123-140.00|86-105-156.00|	156
ENSG00000270084	ENST00000602767	*GAS5-AS1*	*hsa-miR-485-5p*	2	60-81-153.00|467-487-150.00|	153
ENSG00000270084	ENST00000602767	*GAS5-AS1*	*hsa-miR-1537-5p*	1	695-719-151.00|	151
ENSG00000270084	ENST00000602767	*GAS5-AS1*	*hsa-miR-4486*	1	62-77-153.00|	153
ENSG00000270084	ENST00000602767	*GAS5-AS1*	*hsa-miR-1260b*	1	33-51-155.00|	155
ENSG00000270084	ENST00000602767	*GAS5-AS1*	*hsa-miR-548l*	1	508-529-154.00|	154
ENSG00000270084	ENST00000602767	*GAS5-AS1*	*hsa-miR-4796-3p*	1	334-351-150.00|	150
ENSG00000270084	ENST00000602767	*GAS5-AS1*	*hsa-miR-627-5p*	1	469-494-151.00|	151
ENSG00000270084	ENST00000602767	*GAS5-AS1*	*hsa-miR-3929*	1	14-36-153.00|	153
ENSG00000270084	ENST00000602767	*GAS5-AS1*	*hsa-miR-4463*	1	104-121-150.00|	150
ENSG00000270084	ENST00000602767	*GAS5-AS1*	*hsa-miR-890*	1	490-512-154.00|	154
ENSG00000270084	ENST00000602767	*GAS5-AS1*	*hsa-miR-5584-5p*	2	286-307-161.00|71-92-143.00|	161
ENSG00000270084	ENST00000602767	*GAS5-AS1*	*hsa-miR-27b-5p*	1	514-535-163.00|	163
ENSG00000270084	ENST00000602767	*GAS5-AS1*	*hsa-miR-1295b-3p*	1	539-560-150.00|	150
ENSG00000270084	ENST00000602767	*GAS5-AS1*	*hsa-miR-6733-3p*	2	236-255-145.00|789-809-154.00|	154
ENSG00000270084	ENST00000602767	*GAS5-AS1*	*hsa-miR-5680*	1	433-454-166.00|	166

ENSG00000270084	ENST00000602767	*GAS5-AS1*	*hsa-miR-5588-3p*	1	154-172-162.00|	162
ENSG00000270084	ENST00000602767	*GAS5-AS1*	*hsa-miR-624-3p*	1	604-627-152.00|	152
ENSG00000270084	ENST00000602767	*GAS5-AS1*	*hsa-miR-129-5p*	1	767-787-154.00|	154
ENSG00000270084	ENST00000602767	*GAS5-AS1*	*hsa-miR-6813-5p*	1	270-292-157.00|	157
ENSG00000270084	ENST00000602767	*GAS5-AS1*	*hsa-miR-329-5p*	2	366-387-146.00|545-567-160.00|	160
ENSG00000270084	ENST00000602767	*GAS5-AS1*	*hsa-miR-5571-5p*	1	570-591-155.00|	155
ENSG00000270084	ENST00000602767	*GAS5-AS1*	*hsa-miR-374b-3p*	1	195-218-155.00|	155
ENSG00000270084	ENST00000602767	*GAS5-AS1*	*hsa-miR-4261*	1	291-305-156.00|	156
ENSG00000270084	ENST00000602767	*GAS5-AS1*	*hsa-miR-7156-3p*	1	635-657-156.00|	156
ENSG00000270084	ENST00000602767	*GAS5-AS1*	*hsa-miR-3115*	1	617-639-157.00|	157
ENSG00000270084	ENST00000602767	*GAS5-AS1*	*hsa-miR-30b-3p*	1	19-40-151.00|	151
ENSG00000270084	ENST00000602767	*GAS5-AS1*	*hsa-miR-519c-3p*	1	585-607-153.00|	153
ENSG00000270084	ENST00000602767	*GAS5-AS1*	*hsa-miR-6780a-5p*	1	17-40-156.00|	156
ENSG00000270084	ENST00000602767	*GAS5-AS1*	*hsa-miR-122-5p*	1	754-771-155.00|	155
ENSG00000270084	ENST00000602767	*GAS5-AS1*	*hsa-miR-454-3p*	1	584-606-173.00|	173
ENSG00000270084	ENST00000602767	*GAS5-AS1*	*hsa-miR-6835-3p*	1	162-182-160.00|	160
ENSG00000270084	ENST00000602767	*GAS5-AS1*	*hsa-miR-181a-5p*	1	680-703-154.00|	154
ENSG00000270084	ENST00000602767	*GAS5-AS1*	*hsa-miR-205-3p*	1	238-259-162.00|	162
ENSG00000270084	ENST00000602767	*GAS5-AS1*	*hsa-miR-7157-3p*	2	531-552-152.00|221-241-153.00|	153
ENSG00000270084	ENST00000602767	*GAS5-AS1*	*hsa-miR-301a-3p*	1	585-606-153.00|	153
ENSG00000270084	ENST00000602767	*GAS5-AS1*	*hsa-miR-130a-3p*	1	585-606-156.00|	156
ENSG00000270084	ENST00000602767	*GAS5-AS1*	*hsa-miR-876-3p*	1	47-69-151.00|	151
ENSG00000270084	ENST00000602767	*GAS5-AS1*	*hsa-miR-519a-3p*	1	586-607-152.00|	152
ENSG00000270084	ENST00000602767	*GAS5-AS1*	*hsa-miR-509-5p*	1	639-659-167.00|	167
ENSG00000270084	ENST00000602767	*GAS5-AS1*	*hsa-miR-19b-2-5p*	1	185-206-152.00|	152
ENSG00000270084	ENST00000602767	*GAS5-AS1*	*hsa-miR-200a-3p*	1	639-662-155.00|	155
ENSG00000270084	ENST00000602767	*GAS5-AS1*	*hsa-miR-575*	1	636-654-152.00|	152
ENSG00000270084	ENST00000602767	*GAS5-AS1*	*hsa-miR-2052*	1	519-540-153.00|	153
ENSG00000270084	ENST00000602767	*GAS5-AS1*	*hsa-miR-4505*	2	468-485-140.00|61-79-151.00|	151
ENSG00000270084	ENST00000602767	*GAS5-AS1*	*hsa-miR-509-3-5p*	1	637-659-153.00|	153
ENSG00000270084	ENST00000602767	*GAS5-AS1*	*hsa-miR-141-3p*	1	639-662-163.00|	163
ENSG00000270084	ENST00000602767	*GAS5-AS1*	*hsa-miR-1268a*	1	58-75-165.00|	165
ENSG00000270084	ENST00000602767	*GAS5-AS1*	*hsa-miR-7151-5p*	1	756-779-156.00|	156
ENSG00000270084	ENST00000602767	*GAS5-AS1*	*hsa-miR-146a-3p*	1	121-142-155.00|	155
ENSG00000270084	ENST00000602767	*GAS5-AS1*	*hsa-miR-450b-5p*	2	182-204-149.00|766-785-153.00|	153
ENSG00000270084	ENST00000602767	*GAS5-AS1*	*hsa-miR-6514-3p*	1	40-60-163.00|	163
ENSG00000270084	ENST00000602767	*GAS5-AS1*	*hsa-miR-664b-5p*	1	164-187-151.00|	151
ENSG00000270084	ENST00000602767	*GAS5-AS1*	*hsa-miR-6845-5p*	1	271-291-152.00|	152
ENSG00000270084	ENST00000602767	*GAS5-AS1*	*hsa-miR-590-3p*	3	247-266-146.00|624-644-155.00|782-799-146.00|	155

ENSG00000270084	ENST00000602767	*GAS5-AS1*	*hsa-miR-6741-5p*	1	96-117-163.00|	163
ENSG00000270084	ENST00000602767	*GAS5-AS1*	*hsa-miR-7113-5p*	1	289-309-161.00|	161
ENSG00000270084	ENST00000602767	*GAS5-AS1*	*hsa-miR-8080*	1	724-747-156.00|	156
ENSG00000270084	ENST00000602767	*GAS5-AS1*	*hsa-miR-1184*	1	636-658-157.00|	157
ENSG00000270084	ENST00000602767	*GAS5-AS1*	*hsa-miR-432-5p*	1	487-511-176.00|	176
ENSG00000270084	ENST00000602767	*GAS5-AS1*	*hsa-miR-23b-3p*	1	682-705-152.00|	152
ENSG00000270084	ENST00000602767	*GAS5-AS1*	*hsa-miR-8084*	1	440-462-153.00|	153
ENSG00000270084	ENST00000602767	*GAS5-AS1*	*hsa-miR-511-5p*	1	208-226-151.00|	151
ENSG00000270084	ENST00000602767	*GAS5-AS1*	*hsa-miR-23c*	1	683-705-154.00|	154
ENSG00000270084	ENST00000602767	*GAS5-AS1*	*hsa-miR-6776-5p*	1	99-118-156.00|	156
ENSG00000270084	ENST00000602767	*GAS5-AS1*	*hsa-miR-3148*	1	69-90-156.00|	156
ENSG00000270084	ENST00000602767	*GAS5-AS1*	*hsa-miR-5047*	1	226-246-154.00|	154
ENSG00000270084	ENST00000602767	*GAS5-AS1*	*hsa-miR-6089*	1	15-37-168.00|	168
ENSG00000270084	ENST00000602767	*GAS5-AS1*	*hsa-miR-450a-1-3p*	1	400-420-155.00|	155
ENSG00000270084	ENST00000602767	*GAS5-AS1*	*hsa-miR-5787*	3	466-485-141.00|99-120-140.00|61-79-150.00|	150
ENSG00000270084	ENST00000602767	*GAS5-AS1*	*hsa-miR-4711-3p*	1	605-622-152.00|	152
ENSG00000270084	ENST00000602767	*GAS5-AS1*	*hsa-miR-132-3p*	1	388-409-152.00|	152
ENSG00000270084	ENST00000602767	*GAS5-AS1*	*hsa-miR-30d-3p*	1	232-258-152.00|	152
ENSG00000270084	ENST00000602767	*GAS5-AS1*	*hsa-miR-4295*	1	589-606-167.00|	167
ENSG00000270084	ENST00000602767	*GAS5-AS1*	*hsa-miR-4761-5p*	1	605-626-154.00|	154
ENSG00000270084	ENST00000602767	*GAS5-AS1*	*hsa-miR-1273h-5p*	1	20-40-165.00|	165
ENSG00000270084	ENST00000602767	*GAS5-AS1*	*hsa-miR-1268b*	1	56-75-165.00|	165
ENSG00000259887	ENST00000562996	*FIGNL2-DT*	*hsa-miR-4322*	2	329-348-157.00|393-413-155.00|	157
ENSG00000259887	ENST00000562996	*FIGNL2-DT*	*hsa-miR-6787-5p*	3	196-215-151.00|271-290-161.00|164-184-157.00|	161
ENSG00000259887	ENST00000562996	*FIGNL2-DT*	*hsa-miR-6877-5p*	1	203-225-171.00|	171
ENSG00000259887	ENST00000562996	*FIGNL2-DT*	*hsa-miR-6870-5p*	2	97-115-151.00|268-286-157.00|	157
ENSG00000259887	ENST00000562996	*FIGNL2-DT*	*hsa-miR-6829-5p*	2	18-37-162.00|192-210-152.00|	162
ENSG00000259887	ENST00000562996	*FIGNL2-DT*	*hsa-miR-6735-5p*	1	146-172-154.00|	154
ENSG00000259887	ENST00000562996	*FIGNL2-DT*	*hsa-miR-6732-5p*	2	75-94-150.00|89-110-148.00|	150
ENSG00000259887	ENST00000562996	*FIGNL2-DT*	*hsa-miR-331-3p*	1	381-402-150.00|	150
ENSG00000259887	ENST00000562996	*FIGNL2-DT*	*hsa-miR-4488*	2	1-8-140.00|92-109-155.00|	155
ENSG00000259887	ENST00000562996	*FIGNL2-DT*	*hsa-miR-4728-5p*	3	153-175-145.00|91-113-161.00|198-220-149.00|	161
ENSG00000259887	ENST00000562996	*FIGNL2-DT*	*hsa-miR-212-5p*	1	365-393-154.00|	154
ENSG00000259887	ENST00000562996	*FIGNL2-DT*	*hsa-miR-6746-3p*	1	65-87-150.00|	150
ENSG00000259887	ENST00000562996	*FIGNL2-DT*	*hsa-miR-1233-5p*	2	157-177-146.00|117-	165

					139-165.00|	
ENSG00000259887	ENST00000562996	*FIGNL2-DT*	*hsa-miR-1909-3p*	1	75-96-154.00|	154
ENSG00000259887	ENST00000562996	*FIGNL2-DT*	*hsa-miR-4767*	2	286-308-151.00|192-214-145.00|	151
ENSG00000259887	ENST00000562996	*FIGNL2-DT*	*hsa-miR-7850-5p*	1	407-427-153.00|	153
ENSG00000259887	ENST00000562996	*FIGNL2-DT*	*hsa-miR-4632-5p*	2	150-172-159.00|81-104-143.00|	159
ENSG00000259887	ENST00000562996	*FIGNL2-DT*	*hsa-miR-4787-5p*	2	162-182-156.00|19-40-152.00|	156
ENSG00000259887	ENST00000562996	*FIGNL2-DT*	*hsa-miR-6510-5p*	1	76-97-157.00|	157
ENSG00000259887	ENST00000562996	*FIGNL2-DT*	*hsa-miR-8082*	1	398-418-154.00|	154
ENSG00000259887	ENST00000562996	*FIGNL2-DT*	*hsa-miR-7112-5p*	1	83-104-165.00|	165
ENSG00000259887	ENST00000562996	*FIGNL2-DT*	*hsa-miR-4632-3p*	1	65-86-156.00|	156
ENSG00000259887	ENST00000562996	*FIGNL2-DT*	*hsa-miR-6827-5p*	1	118-137-162.00|	162
ENSG00000259887	ENST00000562996	*FIGNL2-DT*	*hsa-miR-6069*	1	323-344-159.00|	159
ENSG00000259887	ENST00000562996	*FIGNL2-DT*	*hsa-miR-6514-5p*	1	397-417-161.00|	161
ENSG00000259887	ENST00000562996	*FIGNL2-DT*	*hsa-miR-941*	1	314-337-168.00|	168
ENSG00000259887	ENST00000562996	*FIGNL2-DT*	*hsa-miR-4481*	1	398-414-150.00|	150
ENSG00000259887	ENST00000562996	*FIGNL2-DT*	*hsa-miR-762*	3	282-302-145.00|216-235-153.00|325-345-153.00|	153
ENSG00000259887	ENST00000562996	*FIGNL2-DT*	*hsa-miR-7704*	1	162-180-153.00|	153
ENSG00000259887	ENST00000562996	*FIGNL2-DT*	*hsa-miR-6781-5p*	4	285-302-146.00|146-166-143.00|160-179-141.00|212-235-157.00|	157
ENSG00000259887	ENST00000562996	*FIGNL2-DT*	*hsa-miR-7111-5p*	2	96-115-156.00|267-286-146.00|	156
ENSG00000259887	ENST00000562996	*FIGNL2-DT*	*hsa-miR-6727-5p*	3	95-118-140.00|290-316-142.00|213-238-152.00|	152
ENSG00000259887	ENST00000562996	*FIGNL2-DT*	*hsa-miR-4265*	2	330-348-153.00|395-413-148.00|	153
ENSG00000259887	ENST00000562996	*FIGNL2-DT*	*hsa-miR-6850-3p*	2	316-335-156.00|60-81-144.00|	156
ENSG00000259887	ENST00000562996	*FIGNL2-DT*	*hsa-miR-1908-3p*	1	59-80-162.00|	162
ENSG00000259887	ENST00000562996	*FIGNL2-DT*	*hsa-miR-6846-5p*	3	19-39-153.00|191-212-145.00|216-237-140.00|	153
ENSG00000259887	ENST00000562996	*FIGNL2-DT*	*hsa-miR-149-3p*	3	209-229-143.00|201-220-161.00|94-113-152.00|	161
ENSG00000259887	ENST00000562996	*FIGNL2-DT*	*hsa-miR-6879-5p*	3	205-226-144.00|83-104-148.00|151-172-176.00|	176
ENSG00000259887	ENST00000562996	*FIGNL2-DT*	*hsa-miR-6785-5p*	3	154-175-140.00|92-113-153.00|198-220-	153

					147.00|	
ENSG00000259887	ENST00000562996	*FIGNL2-DT*	*hsa-miR-6795-5p*	2	93-116-152.00|267-287-143.00|	152
ENSG00000259887	ENST00000562996	*FIGNL2-DT*	*hsa-miR-7854-3p*	1	260-282-158.00|	158
ENSG00000259887	ENST00000562996	*FIGNL2-DT*	*hsa-miR-7158-5p*	1	382-408-156.00|	156
ENSG00000259887	ENST00000562996	*FIGNL2-DT*	*hsa-miR-1908-5p*	4	163-184-146.00|195-215-155.00|269-290-149.00|150-170-151.00|	155
ENSG00000259887	ENST00000562996	*FIGNL2-DT*	*hsa-miR-6816-5p*	2	271-292-158.00|89-108-154.00|	158
ENSG00000259887	ENST00000562996	*FIGNL2-DT*	*hsa-miR-6827-3p*	1	44-63-152.00|	152
ENSG00000259887	ENST00000562996	*FIGNL2-DT*	*hsa-miR-3620-5p*	2	282-303-148.00|324-346-151.00|	151
ENSG00000259887	ENST00000562996	*FIGNL2-DT*	*hsa-miR-663a*	4	195-215-153.00|148-170-155.00|271-290-149.00|163-184-153.00|	155
ENSG00000259887	ENST00000562996	*FIGNL2-DT*	*hsa-miR-3918*	2	148-168-157.00|208-227-140.00|	157
ENSG00000259887	ENST00000562996	*FIGNL2-DT*	*hsa-miR-1184*	1	12-34-150.00|	150
ENSG00000259887	ENST00000562996	*FIGNL2-DT*	*hsa-miR-6883-5p*	2	92-113-153.00|199-220-141.00|	153
ENSG00000259887	ENST00000562996	*FIGNL2-DT*	*hsa-miR-3193*	1	354-375-152.00|	152
ENSG00000259887	ENST00000562996	*FIGNL2-DT*	*hsa-miR-6821-5p*	2	351-373-151.00|121-142-148.00|	151
ENSG00000259887	ENST00000562996	*FIGNL2-DT*	*hsa-miR-6825-5p*	2	264-285-150.00|94-114-149.00|	150
ENSG00000259887	ENST00000562996	*FIGNL2-DT*	*hsa-miR-7843-5p*	1	151-172-159.00|	159
ENSG00000259887	ENST00000562996	*FIGNL2-DT*	*hsa-miR-6741-5p*	2	348-369-160.00|127-146-145.00|	160
ENSG00000259887	ENST00000562996	*FIGNL2-DT*	*hsa-miR-6132*	2	151-169-158.00|83-101-153.00|	158
ENSG00000259887	ENST00000562996	*FIGNL2-DT*	*hsa-miR-4743-5p*	2	211-234-163.00|278-301-140.00|	163
ENSG00000259887	ENST00000562996	*FIGNL2-DT*	*hsa-miR-3180-5p*	1	359-383-151.00|	151
ENSG00000259887	ENST00000562996	*FIGNL2-DT*	*hsa-miR-449c-3p*	1	184-206-151.00|	151
ENSG00000259887	ENST00000562996	*FIGNL2-DT*	*hsa-miR-5698*	2	94-115-142.00|269-286-151.00|	151
ENSG00000259887	ENST00000562996	*FIGNL2-DT*	*hsa-miR-3196*	2	275-292-150.00|90-108-152.00|	152
ENSG00000259887	ENST00000562996	*FIGNL2-DT*	*hsa-miR-1237-5p*	4	198-218-141.00|91-109-156.00|1-8-140.00|155-173-140.00|	156
ENSG00000259887	ENST00000562996	*FIGNL2-DT*	*hsa-miR-4296*	1	332-348-151.00|	151
ENSG00000259887	ENST00000562996	*FIGNL2-DT*	*hsa-miR-182-5p*	1	367-391-157.00|	157

ENSG00000259887	ENST00000562996	*FIGNL2-DT*	*hsa-miR-299-3p*	1	328-349-156.00|	156
ENSG00000259887	ENST00000562996	*FIGNL2-DT*	*hsa-miR-6805-5p*	2	192-213-143.00|89-110-159.00|	159
ENSG00000259887	ENST00000562996	*FIGNL2-DT*	*hsa-miR-1207-5p*	1	150-170-159.00|	159
ENSG00000259887	ENST00000562996	*FIGNL2-DT*	*hsa-miR-2278*	1	77-99-159.00|	159
ENSG00000259887	ENST00000562996	*FIGNL2-DT*	*hsa-miR-1307-3p*	1	167-187-163.00|	163
ENSG00000259887	ENST00000562996	*FIGNL2-DT*	*hsa-miR-6836-5p*	1	149-169-156.00|	156
ENSG00000259887	ENST00000562996	*FIGNL2-DT*	*hsa-miR-4466*	2	302-319-148.00|26-44-151.00|	151
ENSG00000259887	ENST00000562996	*FIGNL2-DT*	*hsa-miR-4745-5p*	1	388-414-154.00|	154
ENSG00000259887	ENST00000562996	*FIGNL2-DT*	*hsa-miR-17-3p*	1	13-34-150.00|	150
ENSG00000259887	ENST00000562996	*FIGNL2-DT*	*hsa-miR-4763-3p*	2	73-97-152.00|148-170-159.00|	159
ENSG00000259887	ENST00000562996	*FIGNL2-DT*	*hsa-miR-6752-5p*	1	87-109-151.00|	151

Notes: (§) **MRE:** miRNA response elements; (*) Max miRANDA score provides assessment of pairing between miRNA sequence with target genes. Bioinformatic tool for making predictions, as it utilizes the most recent miRanda prediction. [**Miranda**, (http://www.microrna.org) applies rules such as seed-site pairing, site context, free-energy, and conservation. Stringent criteria consider pairing score > 150]. (#)Search & analysis was performed in ncFAN v2.0 database (see details in text).

Table S2. DNA methylation landscape of the lncRNA transcripts in gastrointestinal (GI) carcinogenesis retrieved from Lnc2Meth database (*).

**Disease state**	**Published LncRNA symbol**	**Region**	**Pattern**	**Regulatory mechanism**	**Prognostic value**	**Year**	**PubMed (PMID)**
Colorectal Cancer	*ecCEBPA*	*CEBPA* distal promoter	hypomethylation	Trans-Methylation Due to LncRNAs	unknown	2013	24107992
Colorectal Cancer	*H19*	*IGF2/H19* DMR	hypomethylation	Cis-Methylated LncRNAs	unknown	2012	22427002
Colorectal Cancer	*H19*	*H19* DMR	hypomethylation	Cis-Methylated LncRNAs	unknown	2010	19957330
Colorectal Cancer	*LIT1*	*KvDMR1*	hypermethylation	Cis-Methylated LncRNAs	unknown	2006	16965397
Colorectal Cancer	*XIST*	*XIST*	hypomethylation	Cis-Methylated LncRNAs	unknown	2014	25387668
Colorectal Cancer	*XIST*	*XIST* 5' end	demethylation	Cis-Methylated LncRNAs	unknown	2005	16769694
Colorectal Cancer	*ZNF582-AS1*	promoter CpG islands (CGIs) of *ZNF582-AS1*	hypermethylation	Cis-Methylated LncRNAs	yes	2016	27215978
Gastric Cancer	*AK058003*	*SNCG* gene CpG island	methylation	Trans-Methylation Due to LncRNAs	unknown	2014	25499222
Gastric Cancer	*AK123072*	*EGFR* gene CpG island	methylation	Trans-Methylation Due to LncRNAs	unknown	2015	26884908
Gastric Cancer	*GAS5*	*GAS5* promoter	hypermethylation	Cis-Methylated LncRNAs	unknown	2016	27466992
Gastric Cancer	*MEG3*	promoter of the *MEG3*	hypermethylation	Cis-Methylated LncRNAs	unknown	2014	24515776
Gastric Cancer	*MEG3*	DMR of the *MEG3* gene	hypermethylation	Cis-Methylated LncRNAs	unknown	2013	24006224
Gastric Cancer	*SPRY4-IT1*	-	methylation	Cis-Methylated LncRNAs	unknown	2015	26238992
Gastric Cancer	*TP53TG1*	TP53TG1 promoter CpG island	hypermethylation	Cis-Methylated LncRNAs	yes	2016	27821766
Colorectal Neoplasia	*CAHM*	CpG sites in the *CAHM* locus	hypermethylation	Cis-Methylated LncRNAs	unknown	2014	24799664
Colorectal Neoplasia	*H19*	*H19* DMR	methylation	Cis-Methylated LncRNAs	unknown	2010	22121898
Colon Cancer	*H19*	*H19* promoter	demethylation	Cis-Methylated LncRNAs	yes	2017	28130225
Colon Cancer	*Lnc34a*	*miR-34a* promoter	methylation	Trans-Methylation Due to LncRNAs	unknown	2016	27077950
Colon Cancer	*TP53TG1*	*TP53TG1* promoter CpG island	hypermethylation	Cis-Methylated LncRNAs	unknown	2016	27821766
Colon Carcinoma	*TERRA*	*TERRA* CpG-island promoters	methylation	Cis-Methylated LncRNAs	unknown	2009	19850908
Gastrointestinal Stromal Tumor	*HOTAIR*	*PCDH10* promoter CpG islands	methylation	Trans-Methylation Due to LncRNAs	unknown	2016	27659532

Notes: (*) Data searched and retrieved from Lnc2Meth, a manually curated database of regulatory relationships between long non-coding RNAs and DNA methylation associated with human gastrointestinal (GI) carcinogenesis (see details in text).

Table S3. Newly characterized human lncRNA information associated with UC, retrieved and analyzed from various platforms and databases (*).

ID (Ensemb)	Name	Species	Disease name	Chromosome	Exon NO.	Interaction	Interaction target	NCBI accession	Description (interaction)	Description (Function)	PMID
EL0646 (ENSG00000240498)	*CDKN2B-AS1*	Homo sapiens	ulcerative colitis	9	21	U(§)	*EZH2, miR-143-3p*	NR_003529, NR_047532, NR_047543, NR_047535, NR_047537, NR_047534, NR_047536, NR_047538, NR_120536	LncRNA *ANRIL* downregulation in intestinal mucosa correlates with increased disease risk, higher disease activity and elevated proinflammatory cytokines levels, and its change associates with infliximab treatment response in patients with Crohn's Disease. Linear and circular *CDKN2B-AS1* expression is associated with Inflammatory Bowel Disease and participates in intestinal barrier formation. *MALAT1* promotes ulcerative colitis by upregulating *ANRIL*.	ANRIL/EZH2 complex repressed p21/CDKN1A transcription through H3K27 trimethylation of thep21/CDKN1A promoter. CDKN2B-AS1 served as a molecular sponge for miR-143-3p, leading to the derepression of miR-143-3p target SMAD3.	30665494 31207308 32026279 30258009 32305052

EL1183 (ENSG000 00130600)	*H19*	Homo sapiens	ulcerative colitis	11	6	U(§)	*IGF2,let-7, let-7b,miR-138, miR-30a, miR-93.*	NR_131224, NR_131223, NR_002196	*H19* overexpression on intestinal epithelial barrier function and suggests a potential role of *H19* in the d evelopment of UC.	*H19* is an imprinted maternally expressed gene influencing *IGF2* expression, whose transcript is a long noncoding (lncRNA) of unknown biological function harboring the *mir-675*. Acting as a molecular sponge, *H19* inhibits microRNA *let-7*, and acts as a competitive ndogenous RNA against *let-7b*. *miR-138* was identified as a direct target of *H19* and *SOX4*. *miR-30a* negatively regulated *H19* through direct binding. *MiR-93* interacted with either lncRNA *H19* or XBP1s then modulating the inflammatory processes.	27661667 22527881 25399420 32069774 31217890 31026067 31953562.

EL1313 (ENSG00000255733)	*IFNG-AS1*	Homo sapiens	ulcerative colitis	12	5	regulation	*IFNG*	NR_104124, NR_104125.	*IFNG-AS1* as a novel regulator of IFNG inflammatory responses, suggesting the potential importance of ncRNA mechanisms on regulation of IBD-related inflammatory responses. The IBD-associated lncRNA *IFNG-AS1* regulates the balance between inflammatory and anti-inflammatory cytokine production after T-cell stimulation. The effective role of *IFNG-AS1* in many protective actions, including enhancing the expression of INF-γ in the immune response of brucellosis patients, revealed new potential marker, LncRNA *IFNG-AS1* in screening,diagnosis or treatment of brucellosis.	*IFNG-AS1* Enhances IFNG Production in Human Natural Killer Cells.	27492330 31545920 31595441 30661002
EL2382 (ENSG00000251562)	*MALAT1*	Homo sapiens	ulcerative colitis	11	2	U(§)	*CHD1*, *miR-200a*, *miR-200c*	NR_002819, NR_144567, NR_144568.	*MARCH7* interacted with *MALAT1* by *miR-200a*. *MARCH7* may function as a competing endogenous RNA (ceRNA) to regulate the expression of *ATG7* by competing with miR-200a. *MARCH7* regulated TGF-β-smad2/3 pathway by interacting with TGFβR2. *MiR-200c* is bound directly to *MALAT1* as detected by luciferase reporter and qRT-PCR assays. *MALAT1* and *miR-200c* are reciprocally repressed, and TGF-β increased *MALAT1* xpression by inhibiting *miR-200c*. Mechanistically, *MALAT1* was found to interact with NF-κB in the nucleus, thus	*MALAT1* may function as an autonegative feedback regulator of NF-κB to help fine-tune innate immune responses. *MALAT1* promotes ulcerative colitis by upregulating *ANRIL*.	29794480 27693631 27434861 27434861 32026279.

									inhibiting its DNA binding activity and consequently decreasing the production of inflammatory cytokines.		
EL2474 (N/A)	*Mirt2*	Homo sapiens	ulcerative colitis	N/A	N/A	co-expression	*IL-22*	N/A	Mirt2 is downregulated in ulcerative colitis and regulates IL-22 expression in colonic epithelial cells.	*Mirt2* suppresses TNF-α-induced accumulation of *mir-101*, and based on this *Mirt2* exhibits anti-inflammatory roles.	31687015 31513985
EL3745 (ENSG00000253352)	*TUG1*	Homo sapiens	ulcerative colitis	22	4	binding	*miR-142-5p*	NR_152871, NR_110492, NR_002323, NR_110493, NR_152870, NR_152869, NR_152868, NR_152867	*TUG1* attenuated TNF-α-caused apoptosis and inflammatory response in interstitial cells of Cajal by down-regulating *miR-127* and then inactivating NF-κB and Notch pathways. *TUG1* negatively regulated inflammation in ulcerative colitis through *miR-142-p/SOCS1* axis.	*TUG1* could epigenetically suppress *miR-34a* expression via recruiting Enhancer of zeste homolog 2 (EZH2).	30657572 32173492 30551433.

(*) Notes: UC: Ulcerative Colitis. (ENSG):Ensembl accession number of the gene. (--):Ensembl accession number is not available only EL number (1st column). (ELnumber): Accession is shown according to Database Resources of the National Genomics Data Center, China National Center for Bioinformation in 2022, EVLncRNAs2.0. Data from EVLncRNAs2.0, LNCipedia, LncRNADisease v2.0. NONECODE V6. (see details in the text).

Table S4. piRNA expression profile of the top 5 most abundant piRNAs in the samples detected by RNA-seq analysis piRNAQuest V.2 database in colorectal-related carcinogenesis.

GEO Accession	Sample ID	Sample Type	piRNA Id(*)	Pubmed ID
GSE128526	GSM3678996	Colon adenocarcinoma	*hsa_piRNA_41895*<br>*hsa_piRNA_46206*<br>*hsa_piRNA_41896*<br>*hsa_piRNA_32071*<br>*hsa_piRNA_32723*	31110013
GSE128526	GSM3678997	Colon adenocarcinoma	*hsa_piRNA_41895*<br>*hsa_piRNA_46206*<br>*hsa_piRNA_41896*<br>*hsa_piRNA_41033*<br>*hsa_piRNA_33753*	31110013
GSE128526	GSM3678998	Colon adenocarcinoma	*hsa_piRNA_46206*<br>*hsa_piRNA_41895*<br>*hsa_piRNA_41896*<br>*hsa_piRNA_32071*<br>*hsa_piRNA_30964*	31110013
GSE121842	GSM3447676	Colorectal cancer tissue; Tumor stage: T3N1M0 (IIIB)	*hsa_piRNA_29392*<br>*hsa_piRNA_30147*<br>*hsa_piRNA_30146*<br>*hsa_piRNA_424*<br>*hsa_piRNA_18078*	31169949
GSE121842	GSM3447677	Colorectal cancer tissue; Tumor stage: T3N1M0 (IIIB)	*hsa_piRNA_29392*<br>*hsa_piRNA_30147*<br>*hsa_piRNA_30146*<br>*hsa_piRNA_424*<br>*hsa_piRNA_18078*	31169949
GSE121842	GSM3447678	Colorectal cancer tissue; Tumor stage: T4N1bM0 (IIIB)	*hsa_piRNA_29392*<br>*hsa_piRNA_30147*<br>*hsa_piRNA_30146*<br>*hsa_piRNA_424*<br>*hsa_piRNA_18078*	31169949

GSE112492	GSM3071312	Colorectal adenocarcinoma cell line DLD1	*hsa_piRNA_29394*<br>*hsa_piRNA_29392*<br>*hsa_piRNA_30147*<br>*hsa_piRNA_30146*<br>*hsa_piRNA_31003*	32316138
GSE112492	GSM3071313	Colorectal adenocarcinoma cell line DLD1	*hsa_piRNA_29392*<br>*hsa_piRNA_30147*<br>*hsa_piRNA_29394*<br>*hsa_piRNA_36825*<br>*hsa_piRNA_41005*	32316138
GSE112492	GSM3071314	Colorectal adenocarcinoma cell line DLD1	*hsa_piRNA_30146*<br>*hsa_piRNA_36825*<br>*hsa_piRNA_41005*<br>*hsa_piRNA_29392*<br>*hsa_piRNA_30147*	32316138
GSE112492	GSM3071315	Colorectal adenocarcinoma cell line DLD1	*hsa_piRNA_29394*<br>*hsa_piRNA_29392*<br>*hsa_piRNA_30146*<br>*hsa_piRNA_30147*<br>*hsa_piRNA_36825*	32316138
GSE112492	GSM3071316	Colorectal adenocarcinoma cell line HT29	*hsa_piRNA_29392*<br>*hsa_piRNA_30147*<br>*hsa_piRNA_152*<br>*hsa_piRNA_26658*<br>*hsa_piRNA_26659*	32316138
GSE112492	GSM3071317	Colorectal adenocarcinoma cell line HT29	*hsa_piRNA_29392*<br>*hsa_piRNA_30147*<br>*hsa_piRNA_26658*<br>*hsa_piRNA_30146*<br>*hsa_piRNA_424*	32316138
GSE112492	GSM3071318	Colorectal adenocarcinoma cell line HT29	*hsa_piRNA_29392*<br>*hsa_piRNA_30147*<br>*hsa_piRNA_26658*<br>*hsa_piRNA_18078*<br>*hsa_piRNA_26657*	32316138

GSE112492	GSM3071319	Colorectal adenocarcinoma cell line HT29	*hsa_piRNA_29392*<br>*hsa_piRNA_30147*<br>*hsa_piRNA_26658*<br>*hsa_piRNA_26657*<br>*hsa_piRNA_26659*	32316138

Notes: (*) The 5 most abundant piRNAs in the samples detected and included in the list according to the descending order in their expression level (i.e. Counts Per Million (CPM)). The piRNAQuest V.2 database was employed for the analysis and datasets confirmed and retrieved from GEO, in NCBI (see details in text).

.

Table S5. circRNA characteristics from extracellular vesicles (EVs) expressed in colon and small intestine tissue as detected by RNA-seq analysis from exoRBase 2.0 database.

Gene symbol	Gene ID	Gene type	Related circRNA	Detection frequency	Tissue specificity	Specificity score
			**COLON**			
SATB2-AS1	ENSG00000225953.2	lncRNA gene	0	0.134	Colon	3.367
SLC26A3	ENSG00000091138.12	protein coding gene	0	0.051	Colon	3.666
TACR2	ENSG00000075073.14	protein coding gene	0	0.218	Colon	1.801
UBXN10-AS1	ENSG00000225986.1	lncRNA gene	0	0.114	Colon	1.447

Sample type	Tumor mean	Benign mean	Healthy mean	Urine mean	CSF mean	Bile mean	Diff group
			**COLON**				
Blood;Urine;Bile	0.83	0	0.05	1.02	0	2	NA
Blood;Urine;Bile	0.34	0.02	0	0.14	0	0.24	NA
Blood;Urine;CSF;Bile	3.56	0.35	0.54	0.66	1.12	6.74	ML(up)
Blood;Urine	2.43	0.17	1.63	2.27	0	0	NA

Gene symbol	Gene ID	Gene type	Related circRNA	Detection frequency	Tissue specificity	Specificity score
			**SMALL INTESTINE**			
CDHR2	ENSG00000074276.10	protein coding gene	0	0.508	Small Intestine	3.104
CDHR5	ENSG00000099834.18	protein coding gene	0	0.187	Small Intestine	3.223
CEACAM18	ENSG00000213822.6	protein coding gene	0	0.049	Small Intestine	4.301
CEACAM20	ENSG00000273777.4	protein coding gene	0	0.063	Small Intestine	3.972
CHST5	ENSG00000135702.14	protein coding gene	0	0.043	Small Intestine	1.908
CLCA1	ENSG00000016490.15	protein coding gene	0	0.052	Small Intestine	4.007
CLRN3	ENSG00000180745.4	protein coding gene	0	0.069	Small Intestine	2.895
CPO	ENSG00000144410.4	protein coding gene	0	0.069	Small Intestine	2.593
DEFA5	ENSG00000164816.7	protein coding gene	0	0.038	Small Intestine	4.851
DEFA6	ENSG00000164822.4	protein coding gene	0	0.044	Small Intestine	4.851

Sample type	Tumor mean	Benign mean	Healthy mean	Urine mean	CSF mean	Bile mean	Diff group
			**SMALL INTESTINE**				
Blood;Urine;CSF;Bile	4.07	0.7	3.02	2.77	3.52	14.75	HCC(down),CHD(down), KIRC(up),ML(up)
Blood;Urine;CSF;Bile	1.16	0.06	0.47	7.28	0.29	10.59	NA
Blood;Bile	1.88	0.01	0.01	0	0	7.31	NA
Blood;Urine;Bile	1.67	0.02	0.17	0.86	0	0.13	ML(up)
Blood;Bile	0.36	0	0.02	0	0	0.08	NA
Blood;Urine;Bile	0.37	0.03	0.07	0.01	0	1.65	NA

Blood;Urine;Bile	1.13	0.2	0.04	14.03	0	14.69	NA
Blood;Urine;CSF	0.68	0.06	0.46	1.62	1.14	0	NA
Blood;Urine;Bile	3.36	0.06	0	0.04	0	3.16	NA
Blood;Bile	3.5	0.07	0.48	0	0	20.57	NA

Notes: The online biotool exoRBase 2.0 was employed to search and visualize the results under normal conditions (see details in text).

Table S6. circRNA characteristics from extracellular vesicles (EVs) expressed in colorectal cancer (CRC) and detected by RNA-seq analysis from exoRBase 2.0 database.

circID	circBase ID	Genomic position	chromosome	start	end	Strand	Gene symbol	Gene type
			CRC					
exo_circ_00012	NA	chr10:100243698-100250332	chr10	100243698	100250332	-	*PHBP9*	pseudogene
exo_circ_00077	hsa_circ_0019608	chr10:101797980-101807901	chr10	101797980	101807901	-	*OGA*	protein coding gene
exo_circ_00083	hsa_circ_0019612	chr10:101798842-101807901	chr10	101798842	101807901	-	*OGA*	protein coding gene
exo_circ_00105	hsa_circ_0007564	chr10:101889412-101957732	chr10	101889412	101957732	-	*ARMH3*	protein coding gene
exo_circ_00127	NA	chr10:101990551-102002072	chr10	101990551	102002072	-	*ARMH3*	protein coding gene
exo_circ_00139	hsa_circ_0019656	chr10:102023477-102029745	chr10	102023477	102029745	-	*ARMH3*	protein coding gene
exo_circ_00152	hsa_circ_0000257	chr10:102157019-102158214	chr10	102157019	102158214	+	*NOLC1*	protein coding gene
exo_circ_00164	NA	chr10:102362475-102363798	chr10	102362475	102363798	+	*GBF1*	protein coding gene
exo_circ_00183	NA	chr10:102654653-102656385	chr10	102654653	102656385	+	*TRIM8*	protein coding gene
exo_circ_00205	hsa_circ_0019779	chr10:103091564-103094455	chr10	103091564	103094455	-	*NT5C2*	protein coding gene

Detection frequency	Sample type	Tumor mean	Benign mean	Health mean	Urine mean	CSF mean	Bile mean	Diff group
			CRC					
0.04	Blood	9.09	12.36	3.69	0	0	0	CRC(up),KIRC(up)
0.11	Blood;Urine;Bile	22.02	18.78	32.56	30.9	0	38.67	CRC(up),OV(up)
0.129	Blood;Urine	40.02	23.24	34.14	30.79	0	0	CRC(up),ML(up), OV(up),SCLC(up)
0.171	Blood;Urine	39.36	23.34	36.64	26.91	0	0	CRC(up)
0.021	Blood;Bile	4.9	1.05	3.89	0	0	186.22	CRC(up)
0.069	Blood;Urine	16.56	9.26	21.81	19.29	0	0	CRC(up)
0.236	Blood;Urine	68.87	89.16	49.95	54.83	0	0	BRCA(up),CRC(up), GC(up),KIRC(up), ML(up),OV(up), SCLC(up)
0.083	Blood;Urine	21.06	5.87	1.81	11.01	0	0	CRC(up)
0.027	Blood;Urine	5.76	5.57	0	3.76	0	0	CRC(up)
0.168	Blood;Urine;Bile	32.19	29.99	26.59	46.85	0	38.67	CRC(up),SCLC(up)

Notes: The online biotool exoRBase 2.0 wes employed to search and visualize the results for colorectal cancer (CRC) (see details in text).

FIGURES

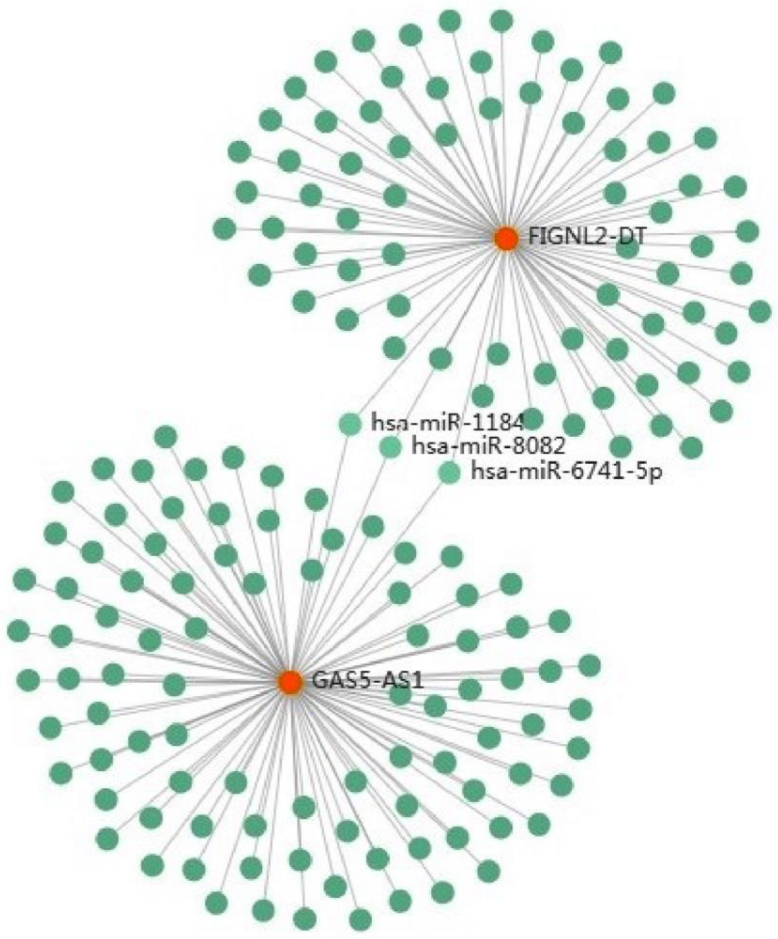

Figure S1. Functional annotation of *FIGNL2-DT* and *GAS5-AS1* lncRNA of the combined network inferred by the functional annotation tool, ncFANs-NET v.2, of the analysed microarray dataset (GSE75459) from patients with Crohn's Disease (CD) (see text). The input ncRNAs source is the gene list of Table S1 and the miRNA interactors that connect FIGNL2-DT and *GAS5-AS1* ncRNAs, are shown between the two hubs (*hsa-miR-1184*, *hsa-miR-8082* and *hsa-miR-6741-5p*). The detailed list of the neighboring miRNAs is presented in Table S1 in the current document of Supplementary Material.

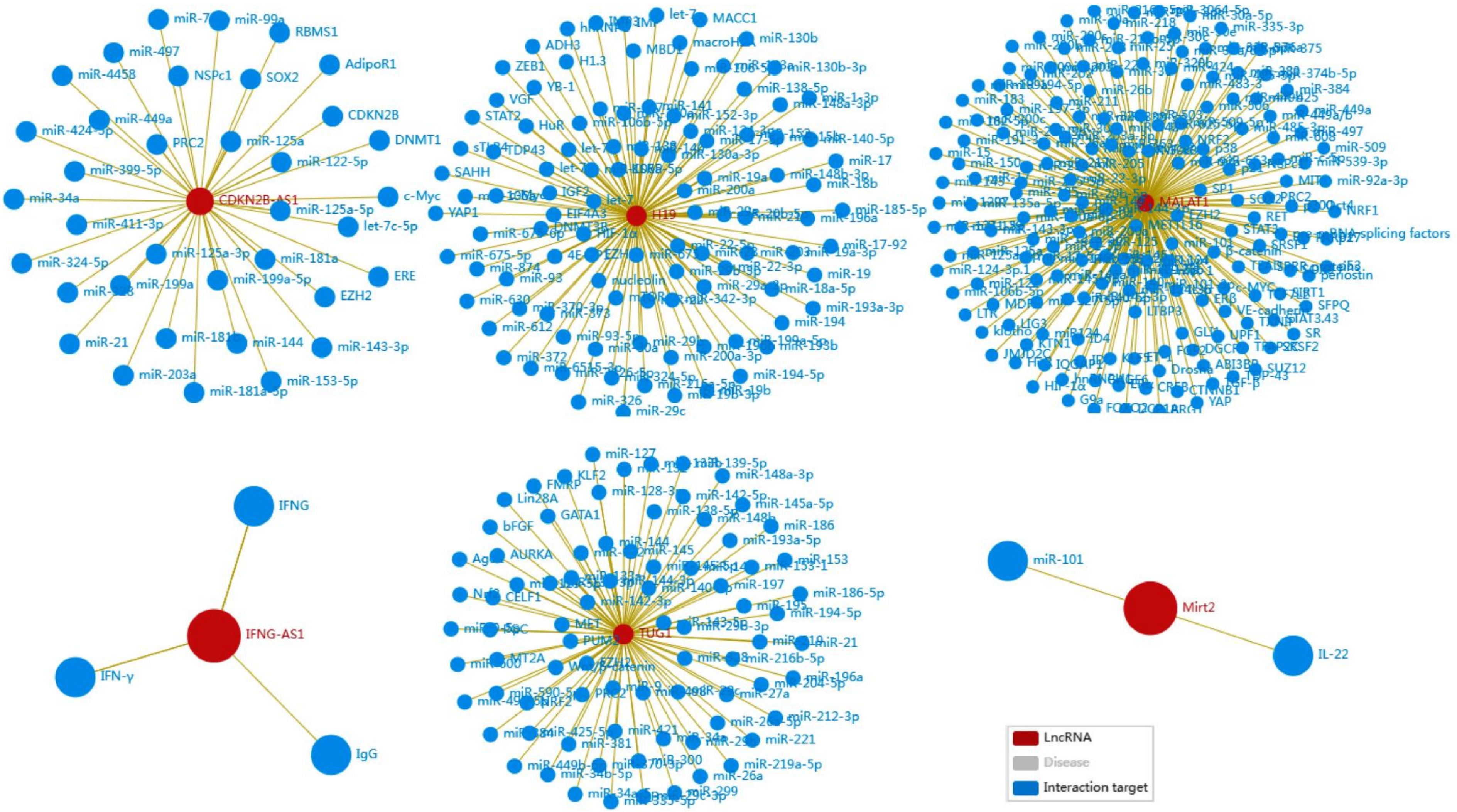

Figure S2. LncRNA-miRNA levels of interaction targets in human disease of UC, using the associated lncRNA information content of EVLncRNAs 2.0 database. Here are presented the lncRNA (red nodes): *CDKN2B-AS1*, *H19*, *IFNG-AS1*, *MALAT1*, *Mirt2* and *TUG1* with their miRNA interaction targets (blue nodes). These ncRNA interaction networks are linked to the Table S3 in the current document of Supplementary Material (see details in the text).

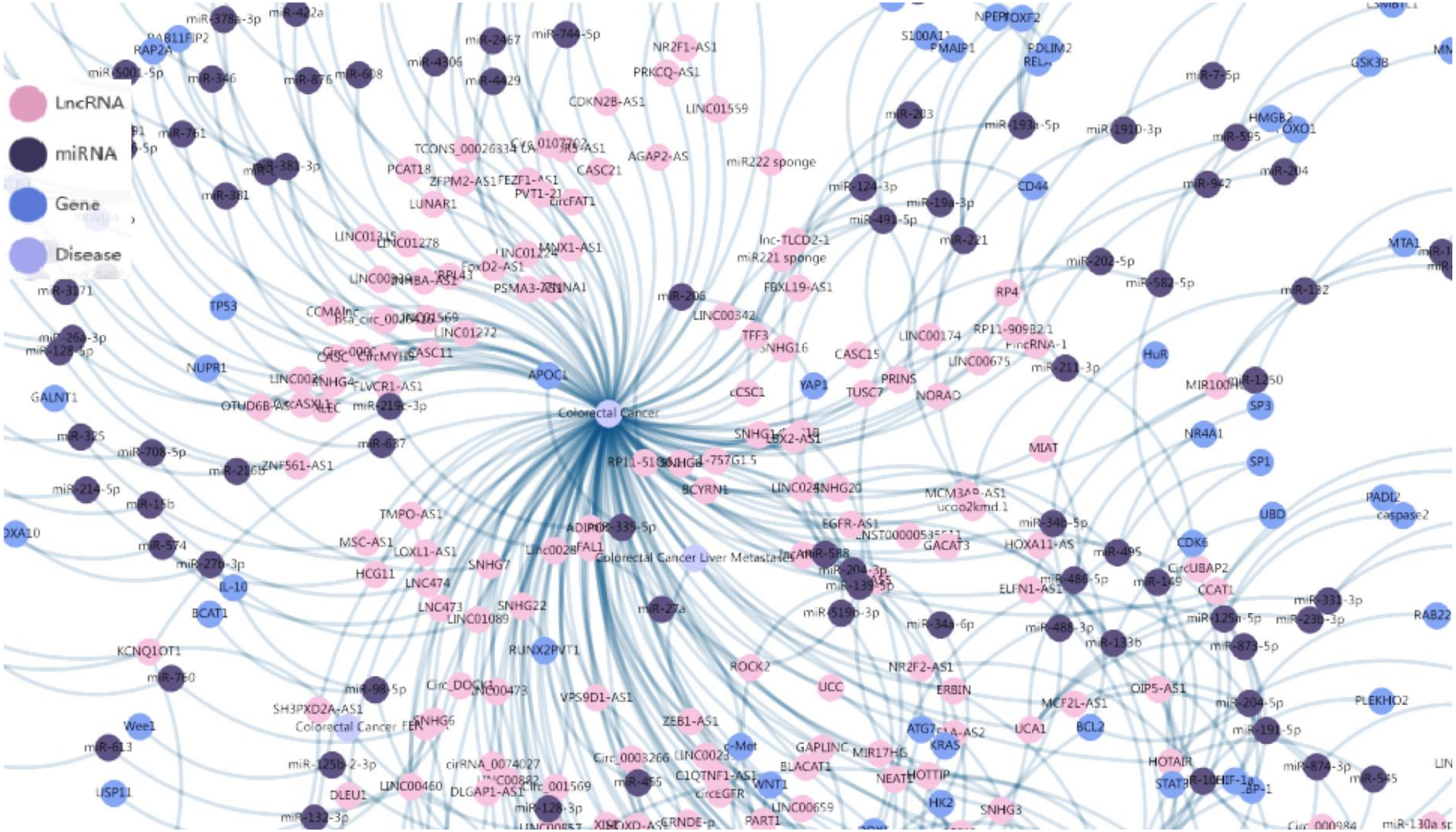

Figure S3. LncRNA-Gene-miRNA-disease network created in LncACTdb 3.0 database from human data (see details in the text), showing in this image significant published targets for GI carcinogenesis in humans. Appendix: The full image shows the dense network with the four types of nodes on the left upper corner (pink, lncRNAs; black, miRNAs; cyan, protein coding genes; violet, disease) as constructed and visualized by the LncACTdb 3.0 database visualization tool. In the center of the network is clearly shown one important disease node of high connectivity, i.e. colorectal cancer (CRC).

<u>LncACT-Network tool settings and input genes used in LncACTdb 3.0 database.</u>

<u>Figure S1</u>

The input symbol/Ensembl IDs for the human IBD-related pathologies that were used in the LncACTdb 3.0 database for the analysis were the following (see details in the text):

lncRNA symbol/Ensembl ID: *H19, CRNDE, SNHG1, DNM30S, SNHG1, MIR99AHG*

mRNA symbol/Ensembl ID: *CTNNB1, AXIN2, CD44, EZH2, CD44, MMP10, WIF1*

miRNAs: *miR-21, miR-155, miR-31, miR-223, miR-29a, let-146a*

Diseases: Colon Adenocarcinoma, Colon Cancer, Colorectal Cancer, Colorectal Cancer Liver Metastases, Colorectal Adenocarcinoma, Ulcerative Colitis.
